# Authority *versus* Independence in Medicine

**Published:** 1910-10-01

**Authors:** 


### AUTHORITY versus INDEPENDENCE IN MEDICINE.

BY A MEDICAL ECLECTIC.

In The Hospital of September 10 " A Medical Heretic " gave some ably expressed advice to the medical student, warning him against the narrowness of outlook consequent upon too ready submission to contemporary authority. His remarks brought back to one a *Vanity Fair* cartoon of the old days, entitled " Medical Orthodoxy." The subject was the late Sir William Broadbent in one of his most vulnerable aspects. Rather unfairly, the artist had chosen to put into the profile the strongest possible suggestion of dogmatic self-assertion, with small hint of the power of achievement which had made his sitter the first medical clinician in the country.

### Tilts at Authority.

However, these skilful strokes at authority are a good thing on the whole, and in these democratic days quite to be expected. They do more, too, than simply delight one as does the discomfiture of the pantomime policeman. " A Medical Heretic " hints that they are especially needed in our profession; he might have added in our nation too. If there be one characteristic of the Englishman that foreign critics are agreed upon it is his respect for law, convention, and authority—his own law, convention, and authority, that is. Thackeray expressed the same thing in an offensive-seeming way when he described us as a nation of snobs. For a snob is a person with excess of reverence for leaders of the established order of things; and although he displeases æsthetically, yet he has his use in keeping up the prestige of the existing necessary administration, and thereby making it difficult for useless schemes of reform to be introduced to derange it; in other words, he defends a tried and workable *status quo* against cranks.

### Pioneers and Snobs.

These last few sentences may appear to contain more than one should attempt to mirror in the steel glass of a medical journal. It is not so, however. Snobbishness forms—does it not?—the antithesis to that quality of temperament common to the revolutionary, to the extreme democrat (who hates all control), and to those who, like perhaps a certain Christian sect, seem sentimentally prejudiced in favour of the second-rate in everything. And these three classes are just the ones generally found ranged on the side of the irregular practitioner of medicine. We may smile at the pettiness of mind that considers a medical man a failure unless he has managed to lodge himself in the Cavendish Square quarter, or that, beguiled by modern bacteriology, cannot conceive of research without a microscope; we may smile at this, but we should also mistrust those who can see nothing but good in any and every medical Ishmael, who are always " agin' " current medical government. Which (speaking generally and with utter absence of intention to give offence) is the more likely patron of the Research Defence Society—a Bishop or a prominent Dissenting preacher, Mr. Arthur Balfour or a leading Fabian? The former in each case, twentyfold. Wherefore, when we are reminded how over much medical orthodoxy may led us to make pretty fools of ourselves by crying down a man in advance of his time, we should wholeheartedly accept the suggestion. No one in the medical profession should have aught but profound respect for the true pioneer—the magician-warrior of scientific imagination who gathers foreknowledge of the truth from the obscurest indications and proves it in the teeth of opposition, dragging lesser minds forward at his chariot wheels; the slightest infusion of this quality is altogether precious. But one may in turn remind " A Medical Heretic " that he may find sheltering behind his eloquent defence some rather undesirable *protégés*.

### The Argument of Self-interest.

He has shrewdly remarked that a powerful liberalising argument is found in the fact that heterodoxy often pays in after life. Of this fact certain people who would engagingly call themselves medical heretics must have the clearest possible perception. A medical consultant in a provincial city told the present writer that the vendor of a widely advertised consumption cure must be making in that district alone a good £2,000 a year. And although it is a common argument with homœopaths that their adherence to an ostracised school proves their disinterestedness, yet the argument is not nearly so strong as it would appear to be. Said a successful general practitioner in a well-known health-resort: " When I came to this town there were already thirty doctors, all allopaths. I, an allopath too, made my practice what you see it. But if I had been a homœopath, like the man who came a year after me, I could have done as well with a quarter of the trouble, because I should have had no competitors."

No, genius and poverty are old acquaintances: genius with a flourishing medicine-packing business (although, as " A Medical Heretic " would doubtless point out, there may be found instances not far from tantamount to this in high places) is not such a likely combination. Genius may take up therapeutical research, but it is much more often ætiological, pathological, or semeiological. Self-interest, or the desire for any sort of notoriety, for obvious reasons sticks to treatment, by preference treatment of a common and serious disease. Genius exerts itself for the sake of the pleasure which comes of discharge of function, not in the hope of remunerative practical applications.

But in spite of these useful and memorable diagnostic points, there will always be uncertainty in some individual cases; and, as has in effect been opined, the young British medical man fresh from five years' academic training may often be usefully reminded of two mottoes on the cover of a little magazine one used occasionally to see on the bookstalls, boldly called " The Crank." The mottoes were these: " A crank is a little thing that makes revolutions." " Take nothing for granted."

